# Cloning and Characterization of Immunological Properties of *Haemophilus influenzae* Enolase

**DOI:** 10.1155/2021/6629824

**Published:** 2021-06-16

**Authors:** Yesenia Osorio-Aguilar, Maria Cristina Gonzalez-Vazquez, Patricia Lozano-Zarain, Ygnacio Martinez-Laguna, Alejandro Carabarin-Lima, Rosa del Carmen Rocha-Gracia

**Affiliations:** ^1^Benemerita Universidad Autonoma de Puebla, Instituto de Ciencias, Centro de Investigaciones en Ciencias Microbiologicas, Posgrado en Microbiologia, Puebla, Puebla, Mexico; ^2^Benemerita Universidad Autonoma de Puebla, Instituto de Ciencias, Licenciatura en Biotecnologia, Mexico

## Abstract

*Haemophilus influenzae* is a common organism of the human upper respiratory tract; this bacterium is responsible of a wide spectrum for respiratory infections and can generate invasive diseases such as meningitis and septicemia. These infections are associated with *H. influenzae* encapsulated serotype b. However, the incidence of invasive disease caused by nontypeable *H. influenzae* (NTHi) has increased in the post-*H. influenzae* serotype b (Hib) vaccine era. Currently, an effective vaccine against NTHi is not available; due to this, it is important to find an antigen capable to confer protection against NTHi infection. In this study, 10 linear B cell epitopes and 13 CTL epitopes and a putative plasminogen-binding motif (_252_FYNKENGMY_260_) and the presence of enolase on the surface of different strains of *H. influenzae* were identified in the enolase sequence of *H. influenzae*. Both *in silico* and experimental results showed that recombinant enolase from *H. influenzae* is immunogenic that could induce a humoral immune response; this was observed mediating the generation of specific polyclonal antibodies anti-rNTHiENO that recognize typeable and nontypeable *H. influenzae* strains. The immunogenic properties and the superficial localization of enolase in *H. influenzae*, important characteristics to be considered as a new candidate for the development of a vaccine, were demonstrated.

## 1. Introduction

*Haemophilus influenzae* is a microorganism that commonly colonizes the upper respiratory tract of both children and adults [1, 2]. This pathogen causes several types of diseases ranging from respiratory tract infections to severe invasive disease, such as meningitis, sepsis, bacteremia, pneumonia, and epiglottitis [3]. Initially, *H. influenzae* strains were classified in capsulated strains (6 capsular serotypes from type Hia through type Hif) and noncapsulated strains named as nontypeable (NTHi) because these are nonreactive with any typing antisera [4]. For a long time, NTHi was the most frequently isolated bacterial pathogens in noninvasive diseases as otitis media and sinusitis in children [1, 5, 6]. However, there have been reported cases of invasive disease caused by NTHi, and moreover, these bacteria have an important role in exacerbation of chronic obstructive pulmonary disease (COPD) in adults; due to this, NTHi is considered as an emerging pathogen [7, 8]. Currently, a vaccine that confers protection against diseases caused by NTHi is not available. A big problem to develop a vaccine is the diversity of NTHi clinical isolates [9], so the challenge is to find a common antigen to all NTHi strains to begin with the development of a vaccine.

Enolase or 2-phospho-D-glycerate hydrolase (EC 4.2.1.11) is a metalloenzyme that catalyzes the dehydration of 2-phosphoglycerate (2-PG) to phosphoenolpyruvate (PEP) in the glycolytic way, requires ion magnesium (Mg^2+^) for their correct function, and is conserved in all eukaryotic and prokaryotic cells [10, 11]. However, this enzyme is considered as moonlighting protein with activity as a virulence factor associated with the surface of pathogens [12] and importantly used as potential immunogen against several bacteria such as *Streptococcus iniae* [13], *Staphylococcus aureus* [14], and *Vibrio parahaemolyticus* [15], showing specificity and capacity to confer protection to experimental infected model.

Although several enolases have been studied with potential as immunogens, the role of enolase in NTHi and their immunogenicity is understood. In this study, we analyzed the immunogenic properties of NTHi enolase and determined the potential to develop an efficient vaccine.

## 2. Material and Methods

### 2.1. Bacterial Strains and Growing Conditions

Four strains (two typeable and two nontypeable) *of H. influenzae* are described as follows: HibBUAPNAN (type b strain, isolated from cerebrospinal fluid from a case of pediatric meningitis), NTHiBUAP96 (strain nontypeable isolated from the middle ear of a case of pediatric otitis media), HiNTBUAPPAU (strain nontypeable isolated from the middle ear of a case of adult otitis media), and HibATCC33930 (strain ATCC of *H. influenzae* type b isolated from cerebrospinal fluid) [16] were cultured in brain-heart infusion (BHI) agar medium (Bioxon de Mexico) supplemented with 5% Fildes (peptic digest of sheep blood that supplies the X (haemin) and V (nicotinamide adenine dinucleotide, NAD), factors necessary for the adequate growth of *H. influenzae*). The strains were incubated at 37°C, 24 h, in a humid atmosphere containing 5% CO_2_. The *E. coli* DH5*α* strain [genetic characteristics: F-*Φ*80*lac*Z*Δ*M15 *Δ*(*lac*ZYA-*arg*F) U169 *rec*A1 *end*A1 *hsd*R17 (r_k_^−^, m_k_^+^) *pho*A *sup*E44 *thi*-1 *gyr*A96 *rel*A1 *λ*-, and BL21 (DE3) pLysS: F^−^, *omp*T *hsd*SB (r_B_^−^m_B_^−^) *gal dcm* (DE3) pLysS (Cam^R^)]; both from Invitrogen™ were grown in Luria-Bertani plates or in Luria-Bertani broth (Bioxon de Mexico) and incubated at 37°C for 24 h under aerobic conditions. Where appropriate, media were supplemented with chloramphenicol at 34 mg mL^−1^ and/or with ampicillin at 100 mg mL^−1^.

### 2.2. Hi*eno* Gene Amplification and Sequencing

The DNA fragment that codes for *Haemophilus influenzae* enolase (Hi*eno*) was amplified from purified genomic DNA of four *H. influenzae* strains (HibBUAPNAN, NTHiBUAP96, HiNTBUAPPAU, and HibATCC33930) by polymerase chain reaction (PCR) using a forward primer (5′-*CTCGAG*ATGGCAAAAATCGTTAAAGTGATTG-3′) and a reverse primer (5′-*GGTAC*CCTTGACCTTTAACCGCTTTTAAG-3′) that introduced both XhoI and KpnI restriction sites (italicized), respectively.

The PCR reaction carried out in a volume of 25 *μ*L contained 1 *μ*g of template, 2 mM of forward and reverse primers, 2 mM MgCl, 0.2 mM of each dNTP, 1X PCR buffer, and 2.5 U of Taq DNA polymerase (NEB, MA, USA). The amplification conditions were 95°C for 1 min, 58°C for 1 min, and 72°C for 2 min for 30 cycles, and 72°C for 10 min. Subsequently, the PCR products of one typeable and one nontypeable strain were cloned in pCR®2.1-TOPO vector (LacZ*α* operon, multiple cloning site, T7 promoter, f1 origin, kanamycin and ampicillin resistance, and pUC origin, Invitrogen™ by Life Technologies, Carlsbad, CA, USA). The resulting plasmids (TOPO::HibBUAPNAN*eno* and TOPO::NTHiBUAP96*eno*) were transformed in *Escherichia coli* DH5*α* cells, and the recombinant positive clones were picked up and later sequenced. The sequence corresponding to NTHiBUAP96*eno* (NTHiENO) was submitted to GenBank (access number: MF405339.1).

### 2.3. Bioinformatics Analysis

From the database of National Center for Biotechnology Information (NCBI), the sequences of enolase of different microorganisms which presented greater homology with Hi*eno* by Basic Local Alignment Sequence Tool (http://www.ncbi.nlm.nih.gov/BLAST), as well as the sequences where enolase has been used as a vaccine, were obtained. Amino acid sequences were aligned using ClustalW (http://www.ebi.ac.uk/Tools/msa/clustalw2/), and neighbor-joining analysis was performed employing the MEGA X package [17].

The enolase human sequence was used as an outgroup. Gaps were treated as pairwise deletions; amino acid distances were calculated using the Poisson model, and branch support was estimated using bootstrap analysis (1000 bootstraps). Furthermore, the NTHiENO sequence was analyzed to calculate its molecular weight; isoelectric point (pI) and stability index were assessed using ExPASy ProtParam server (http://ca.expasy.org/tools/protparam/html), posttranslation modification sites by MotifScan (http://myhits.isb-sib.ch/cgi-bin/motif_scan), functional domain and key sites by InterProScan (http://www.ebi.ac.uk/InterProScan/), transmembranal region and topology structure by TMpred (http://www.ch.embnet.org/software/TMPRED_form.html), and secondary structure and disulfide bond by PredictProtein (http://www.predictprotein.org/), and linear B cell epitopes were predicted using BepiPred 2.0 Server (http://www.cbs.dtu.dk/services/BepiPred/) and CTL epitopes by NetCTL 1.2 Server (http://www.cbs.dtu.dk/services/NetCTL/). Proteasomal cleavage sites in CTL epitopes were predicted by NetChop 3.1 server (http://www.cbs.dtu.dk/services/NetChop/); on the other hand, the prediction of antigenicity of NTHiENO was predicted using VaxiJen v.2.0 server (http://www.ddg-pharmfac.net/vaxijen/scripts/VaxiJen_scripts/VaxiJen3.pl) and the allergenicity was made by AllergenFP v.1.0 (https://ddg-pharmfac.net/AllergenFP/feedback.py).

### 2.4. Subcloning, Expression, and Purification of HiENO

The Hi*eno* DNA sequence was digested from TOPO::NTHiBUAP96*eno* and TOPO:: HibBUAPNAN*eno* plasmids by double enzyme digestion with XhoI and KpnI and subcloned into the expression vector pRSET-A (overexpression vector, Amp^r^, and 6x His, Invitrogen™ by Life Technologies, Carlsbad, CA, USA), obtaining the plasmids pRSET-A::HiNTBUAP96*eno* and pRSET-A::HibBUAPNAN*eno*; the correct subcloning was confirmed by restriction enzyme analysis and sequencing (results not shown). Since the enolase sequences of the typeable and nontypeable strains present a high degree of homology, approximately of 99%, we decided to obtain only the recombinant enolase of NTHiBUAP96 strain (rNTHiENO) as described below.

*E. coli* BL21(DE3) pLysS cell containing the plasmid pRSET-A::HiNTBUAP96*eno* was grown to mid-logarithmic phase at 37°C, in Luria-Bertani (LB) medium supplemented with ampicillin (100 mg mL^−1^) and chloramphenicol (34 mg mL^−1^); subsequently, isopropyl *β*-D-1-thiogalactopyranoside (IPTG) was added at a final concentration of 1 mM to induce overexpression of the recombinant protein (rNTHiENO); the culture was kept 3 hours in constant shaking at 37°C. After induced expression, the bacterial cells were collected by centrifugation at 8000 × *g* for 5 min at 4°C. The final pellet was resuspended in 20 mM Tris-HCl (pH 8.0), 500 mM NaCl, and 5 mM imidazole and disrupted by sonication, followed by centrifugation at 8000 × *g* for 10 min at 4°C; the supernatant was obtained, and the recombinant protein (rNTHiENO) was purified through Ni-NTA-agarose affinity chromatography (Qiagen, Hilden, Germany) according to the manufacturer's instructions. The rNTHiENO was eluted with elution buffer 20 mM Tris-HCl (pH = 8.0), 500 mM NaCl, and 1 M imidazole. Finally, purified rNTHiENO was resolved on 12% sodium dodecyl sulfate-polyacrylamide gel electrophoresis (SDS-PAGE) to analyze its expression. Aliquots of the purified protein were stored at −80°C, and the protein concentration was determined using the Bradford protein assay.

### 2.5. Identification of rNTHiENO by Western Blot Analysis

Purified recombinant rNTHiENO (10 *μ*g) was resolved on a 12% SDS-PAGE and electrotransferred onto nitrocellulose membrane at 15 V for 40 min. The membranes were blocked with a blocking buffer (5% skimmed milk (*w*/*v* in phosphate-buffered saline (PBS))) for 1 h at 37°C under gentle agitation, followed by three washes with PBS containing 0.05% Tween-20 (PBST), and then were incubated overnight at 4°C with antihistidine monoclonal antibody (Thermo Fisher Scientific) at 1 : 5000 dilution in 2% skimmed milk-PBS.

After the washing procedure, the membranes were subsequently incubated with anti-mouse IgG alkaline phosphatase-conjugated secondary antibody (Novex® by Life Technologies) diluted at 1 : 5000 for 1 h at 37°C, and the signal was revealed with nitro blue tetrazolium (NBT) chloride and 5-bromo-4-chloro-3′-indolyphosphate p-toluidine salt (BCIP) (Thermo Fisher Scientific).

### 2.6. Generation of rNTHiENO Polyclonal Antibodies

For the immunization scheme, eight New Zealand rabbits were randomly assigned to the control or immunized groups of four animals each. The rabbits were bled to collect preimmune sera; later, they were immunized with 100 *μ*g of rNTHiENO with complete Freund's adjuvant (CFA) (Sigma) by intramuscular puncture, followed by three boosters with the same concentration of recombinant protein emulsified with incomplete Freund's adjuvant (IFA) (Sigma) in a period of fifteen days each. The control group received PBS/adjuvant in the same schedule as the immunized rabbits; at the end of the immunization scheme, the rabbits were bled to obtain the hyperimmune sera. The rabbits were housed in a controlled environment and managed according to the National Institutes of Health *Guide to the Care and Use of Experimental Animals* and followed the guidelines of the Norma Official Mexicana *Guide for the Care and Use of Laboratory Animals* (NOM-062-ZOO-1999) and with the approval of the Claude Bernal Animal Care and Use Committee of the Benemerita Universidad Autonoma de Puebla.

### 2.7. Generation of *H. influenzae* Polyclonal Antibodies

Polyclonal antiserums against the NTHiBUAP96 and HibBUAPNAN were obtained as follows: a culture of either bacteria NTHi or Hib was recovered in sterile PBS until 10^9^ CFU/mL was obtained and an aliquot with 0.3 mL was subcutaneously injected into two New Zealand rabbits (2.5-3.0 kg) emulsified with an equal volume of complete Freund's adjuvant. The animals received four booster injections (on days 14, 22, 30, and 38 after initial priming immunization) in the presence of incomplete Freund's adjuvant. Antibody titers were examined by western blot at 0, 2, 3, and 4 weeks after the last immunization, and the animals were sedated and killed by cardiac exsanguination at the end of the fourth week and the serum collected was stored at −20°C until use.

### 2.8. Enolase Identification by Indirect Immunofluorescence (IFI)

For microscopic examinations, bacteria (NTHiBUAP96, NTHiBUAPPAU, HibBUAPNAN, and HibATCC33930) were grown overnight on cultures in BHI supplemented with Fildes at 37°C. Later, a drop containing two to three colonies was resuspended in 10 *μ*L of PBS and was pipette onto a glass slide, dried and fixed with 4% paraformaldehyde (PFA) in phosphate-buffered saline (PBS) pH 7.4 for 30 min at room temperature. Slides were washed two times in PBS. After that, samples were incubated all night at 4°C with anti-rNTHiENO polyclonal antiserum (dilution 1 : 50 in 0.1% bovine serum albumin in PBS) and another 60 min at 37°C with anti-rabbit FITC-labeled IgG purchased from Zymax™ (dilution 1 : 250 in BSA-PBS). The slides were then washed with PBS, and 8 *μ*L of VECTASHIELD Antifade Mounting Medium with DAPI (Vector Laboratories) was added to the sample before topping with a coverslip. All preparations were examined by epifluorescence microscopy using a Motic BA410E microscope with a 60x or 100x oil immersion objectives. Ten to twenty fields of each sample were acquired using a Digital WiFi Microscope Camera Moticam X2 (Motic). Images were analyzed and processed using Motic camera software.

### 2.9. Wild Enolase Identification by Western Blot

Briefly, *H. influenzae* strains (typeable and nontypeable) were harvested from cultures and resuspended in lysis buffer (10 mM Tris-HCl, pH 7.5; 5 mM EDTA; 1% Nonidet P-40; 1 mM phenylmethanesulfonyl fluoride; 10 mg/mL aprotinin; 50 U/L Trasylol; 10 mg/mL leupeptin) by repeated freezing and thawing cycles. Lysates were cleared by centrifugation (30 min, 4°C at 14000 × *g*), and the supernatants were collected and resolved by 12% sodium dodecyl sulfate-polyacrylamide gel electrophoresis (SDS-PAGE, 10 *μ*g per lane) or purified rNTHiENO (10 *μ*g per lane). Proteins were electrotransferred onto nitrocellulose membranes at 15 V for 40 min. The membranes were blocked with a blocking buffer (5% skimmed milk (*w*/*v* in phosphate-buffered saline (PBS))) for 1 h at 37°C, washed three times with PBS containing 0.05% Tween-20 (PBS-T), and then incubated overnight at 4°C with anti-rNTHiENO polyclonal antibodies (1 : 1000 dilution in 2% skimmed milk-PBS). For the titration assay, 10 *μ*g per lane of rNTHiENO was migrated and electrotransferred; after that, different dilutions of the anti-rNTHiENO polyclonal antiserum were performed (1 : 1000; 1 : 10000; 1 : 20000; 1 : 50000; 1 : 40000 in 2% skimmed milk-PBS). The negative control consisted of a pool of serum from different healthy rabbits diluted at 1 : 500 in 2% skimmed milk-PBS. After washing, the membranes were incubated with secondary antibody conjugated with alkaline phosphatase (Millipore) for 1 h at 37°C and diluted at 1 : 5000 in 2% skimmed milk-PBS. The blots were visualized with nitro blue tetrazolium (NBT) chloride and 5-bromo-4-chloro-3′-indolyphosphate p-toluidine salt (BCIP) (Thermo Scientific).

## 3. Results

### 3.1. Obtaining the DNA Fragment That Codes for Hi*eno*

**Figure 1:**
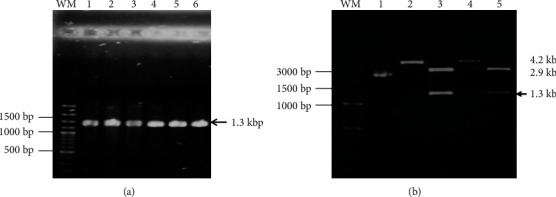
DNA fragment encoding Hi*eno*, amplification, and subcloning. (a) Agarose gel stained with ethidium bromide revealing the 1.3 kbp-sized PCR amplification products (arrow): HibATCC33930 (lane 1), NTHiBUAPPAU (lane 2), HibBUAPNAN (lane 3), amplified obtained using TOPO::HibBUAPNAN*eno* plasmid as a template (lane 4), NTHiBUAP96 (lane 5), and amplified obtained using TOPO::NTHiBUAP96*eno* plasmid as a template (lane 6). (b) Design and construction of pRSET-A::HiENO plasmid. Lane 1: pRSET-A/XhoI digested (2.9 kbp). Lane 2: pRSET-A::HibBUAPNAN plasmid/XhoI. Lane 3: pRSET-A::HibBUAPNAN plasmid digested with XhoI and KpnI. Lane 4: pRSET-A::NTHiBUAP96 plasmid/Xho. Lane 5: pRSET-A::NTHiBUAP96 plasmid digested with XhoI and KpnI enzymes in a double restriction reaction. The product of 1.3 kbp corresponding to Hi*eno* gene is observed (arrow).

From purified DNA genomic of several *H. influenzae* strains typeable and nontypeable, the encoding region to enolase of NTHiBUAP96 was ligated to the pRSET-A vector. PCR reaction was performed with the aim to obtain the DNA fragment that codes for Hi*eno*. A band of 1.3 kbp was obtained in all strains assayed HibATCC33930, HiNTBUAPPAU, HibBUAPNAN, and HiNTBUAP96 (Figure 1(a)). After that, the product corresponding to fragment that codes for Hi*eno* of HibBUAPNAN and HiNTBUAP96 was subcloned into pRSET-A plasmid (Figure 1(b)), and the construction result was sequenced to confirm that the PCR product inserted corresponds to fragment that codes for Hi*eno.*

The complete enolase sequences of both HibBUAPNAN and NTHiBUAP96 strains were obtained, which have 99.54% homology. NTHiBUAPPAU and HibBUAP33930 just obtained the partial sequence. The sequences obtained in this study were compared with the enolase of typeable and nontypeable *H. influenzae* strains that are available in the database of National Center for Biotechnology Information (NCBI); the results showed >99% homology. These results indicate that the enolase sequences in all *H. influenzae* strains have no significant difference (data not shown). The sequence corresponding to NTHiBUAP96*eno* (NTHiENO) was submitted to GenBank (access number: MF405339.1).

The traduced sequence reports that NTHiENO (HiNTBUAP96) has 437 amino acids with a theoretical molecular weight and pI of 46.30 kDa and 5.03, respectively. The half-life was estimated at 30 h, and the protein was classified as stable with a stability index (II) of 28.60 (a value below 40 is considered stable).

The *in silico* analyses were made with the enolase sequence of both strains, but the results yielded the same data; for this reason, the results are only shown for the enolase of the NTHiBUAP96 strain.

### 3.2. Bioinformatics Assays with the NTHiENO Sequence

#### 3.2.1. Identification of the Catalytic and Metal-Binding Site

**Figure 2:**
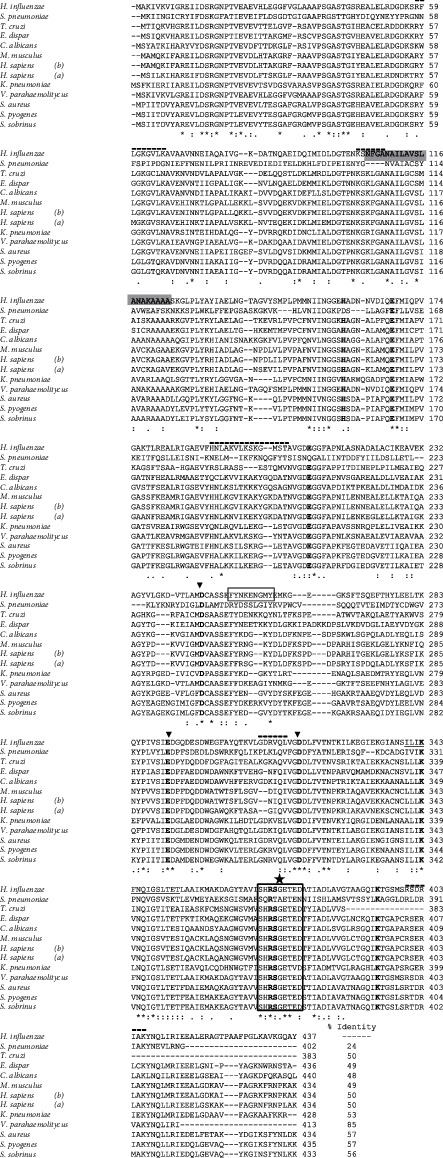
Comparative analysis by alignment of the amino acid sequences of several enolases: *Haemophilus influenzae* NTHiENO (ASO96723.1), *Streptococcus pneumoniae* (CTN02995.1), *Trypanosoma cruzi* (AGR66223.1), *Entamoeba dispar* (EDR27966.1), *Candida albicans* (AAA71939.1), *Mus musculus* (CAA40913.1), *Homo sapiens β*-enolase (NP_001967.3), *Homo sapiens α*-enolase (NP_001419.1), *Klebsiella pneumoniae* (SAW34188.1), *Vibrio parahaemolyticus* (KZW93652.1), *Staphylococcus aureus* (BBA23431.1), *Streptococcus pyogenes* (AKI77548.1), *Streptococcus sobrinus* (CAD60544.1); parentheses show GenBank accession numbers. The conserved amino acids in all sequences are labeled with asterisks; the conservative and semiconservative substitutions are labeled with two and one points, respectively. The dashed lines shown gaps introduced between the sequences. The residues involved in Mg^2+^ binding are shown in bold and with arrowhead, those forming the enolase signature are underlined, and the motifs for substrate binding are indicated with letters in bold. The putative plasminogen-binding site is boxed. The transmembranal domain is indicated in shadowed gray. The enolase “fingerprint” motif is boxed and shown with star. RNA-binding motifs are indicated with dashed bold lines over the motive. The percentage of sequence identity between NTHiENO and the enolases from other organisms is indicated at the end of figure.

With the aim to identify if NTHiENO sequence contains the characteristic motif of enolases, several sequences were downloaded of the DataBank (PubMed) and alignments were done. HiENO sequence shows a strong identity as well as with the heterologous sequence of *Vibrio parahaemolitycus* (85%). With other enolase sequences (bacteria, parasites, and eucarya organism), HiENO showed identity percent ranging from 57% to 24% (Figure 2). The alignment between this enolase's sequences revealed that NTHiENO presents the motifs of enolase as Mg^2+^-binding sites, substrate-binding sites, the sequence considered as enolase signature, the fingerprint motif, putative plasminogen-binding motif, and exRNA-binding sites (Table 1).

#### 3.2.2. Identification of the Plasminogen-Binding Site

With the aim to localize a putative site for interaction with plasminogen in the NTHiENO sequence, an alignment was realized and a putative plasminogen-binding site (_252_FYNKENGMY_260_) was identified; this site really is present in several sequences of parasites and bacteria, which is inclusive and had an important percent of homology (55.5%) with the characterized plasminogen-binding site in *S. pneumoniae* (Table 1). This result indicates that *H. influenzae enolase* could bind to the human plasminogen through this putative plasminogen-binding motif as has been demonstrated in other models.

#### 3.2.3. Identification of Host-Derived Extracellular RNA- (exRNA-) Binding Sites

Recently, it has been demonstrated in *S. pneumoniae* the presence of six exRNA-binding sites in the enolase protein; moreover, these motifs are important for the adhesion to and its internalization into different cells [18]. Therefore, in this study, the putative exRNA-binding sites in NTHiENO sequence were identified. NTHiENO shows five out of six exRNA-binding sites with an identity between 44 and 85% (Table 1); importantly, they conserved amino acids with positive charge (lysine and arginine) presumptively implicated in the interaction with exRNA. Only one motif was not present in NTHiENO corresponding to _432_LKK_434_ motif, located in the C-terminal region of *S. pneumoniae* enolase. Although it is unknown if *H. influenzae* interact with exRNA, there is a possibility that HiENO may interact with the host-derived exRNA, favoring adhesion to the host's cells. However, experimental studies are required to verify this.

#### 3.2.4. Phylogenetic Analysis

**Figure 3:**
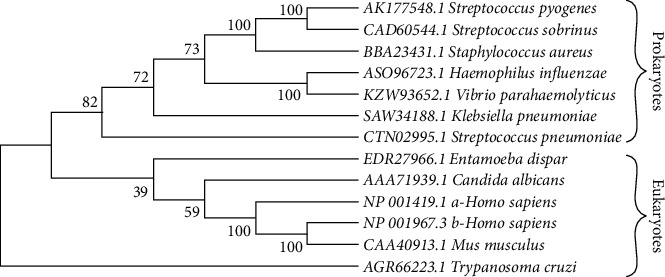
Evolutionary relationships of NTHiENO. The evolutionary history was inferred using the neighbor-joining method [56]. The bootstrap consensus tree inferred from 1000 replicates [57] is taken to represent the evolutionary history of the taxa analyzed [57]. Branches corresponding to partitions reproduced in less than 50% bootstrap replicates are collapsed. The percentages of replicate trees in which the associated taxa clustered together in the bootstrap test (1000 replicates) are shown next to the branches [57]. The evolutionary distances were computed using the Poisson correction method [58] and are in the units of the number of amino acid substitutions per site. This analysis involved 13 amino acid sequences. All ambiguous positions were removed for each sequence pair (pairwise deletion option). There were a total of 454 positions in the final dataset. Evolutionary analyses were conducted in MEGA X [17].

The phylogenetic analysis showed that two major clades are grouped, one corresponding to eukaryotic (support value 91%) and the other one to prokaryotic (support value 63%) organism. Furthermore, NTHiENO is partaking of the node with *V. parahaemolyticus* (Figure 3).

#### 3.2.5. Predicted Epitopes for B and T Cells

**Figure 4:**
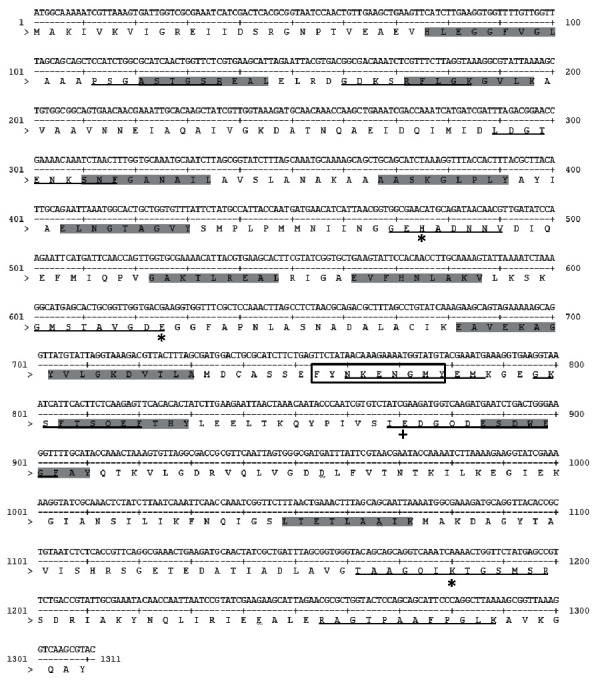
Predicted NTHiENO B and T cell epitopes. The coding region of NTHiENO gene and its translated protein sequence are shown; linear B cell epitopes are underlined; CTL epitopes are shadowed in gray. An asterisk indicates the amino acids involved in the catalytic site (H_159_, E_209_, and K_394_) and with a plus sign and the amino acid involved in binding to Mg^2+^.

With the NTHiENO amino acids, traduced sequences realized predictions to determine if this protein could be immunogenic. The bioinformatics assays predicted the presence of 10 linear B cell epitopes and 13 CTL epitopes conserved into several HLA supertype alleles (Figure 4). The 13 CTL epitopes were examined for the presence of proteasomal cleavage sites using NetChop 3.1 program. All the epitopes displayed several cleavage sites to be processed by the proteasome; this processing by cytosolic pathway is essential for a peptide to act as CTL epitope. Further, four predicted B cell epitopes have overlapped with CTL epitopes (Table 2).

#### 3.2.6. Prediction of Antigenicity and Allergenicity

The antigenicity prediction results by VaxiJen v.2.0 showed a value of 0.4828 (probable antigen); in turn, AllergenFP v.1.0 classifies it as a nonallergen protein; these *in silico* results indicate that NTHiENO is antigenic and should not cause an allergic reaction inside of the body.

#### 3.2.7. Predicted a Transmembrane (TM) Domain

With the aim to know if *H. influenzae* enolase could be in surface, a bioinformatics assay was realized. These studies showed that enolase sequences have a predicted transmembranal domain in the region comprised by amino acids Asn_105_-Ala_124_. Through this transmembranal domain, enolase could be anchoring to the cell surface in *H. influenzae* (Supplementary Figure 1).

### 3.3. Expression and Purification of rNTHiENO

**Figure 5:**
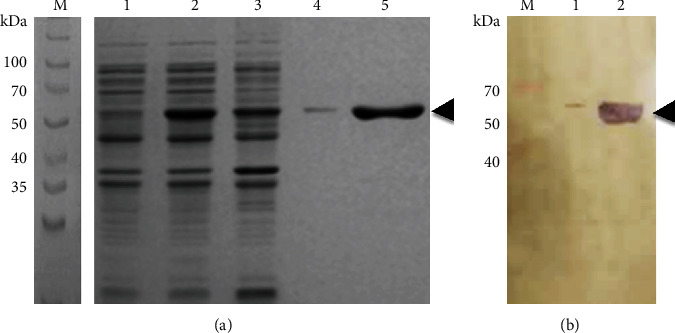
Purification and identification of rNTHiENO. Protein was resolved by SDS-PAGE on 12% polyacrylamide gel and stained with Coomassie blue. (a) Lane M: molecular weight marker. Lane 1: *E. coli* Bl21 cell containing the pRSET-A::NTHiENO without IPTG induction. Lane 2: *E. coli* Bl21 cell containing the pRSET-A::NTHiENO with IPTG induction. Lane 3: insoluble pellet. Lane 4: washing material. Lane 5: the purified rNTHiENO. Identification of rNTHiENO by western blot using a monoclonal antibody anti-His Tag. (b) Lane M: molecular weight marker. Lane 1: pRSET-A::NTHiENO in *E. coli* BL21, noninduced. Lane 2: purified rNTHiENO. The band corresponding to rNTHiENO (52.0 kDa) is indicated with an arrow.

The enolase encoding region of NTHiBUAP96 was ligated to the pRSET-A vector and expressed as a His-tag fusion protein in *E. coli* BL21 (DE3) pLysS cells. The purified protein had a predicted size of approximately 52 kDa, which includes an approximately 6 kDa peptide corresponding to tag fusion (Figure 5(a), lane 5). Then, the purified *H. influenzae* enolase was recognized as a single band (approximately 52 kDa) by western blot assays using a monoclonal anti-His antibody (Figure 5(b), lane 2). A slight signal was identified in extract of *E. coli* Bl21 with the noninduced recombinant plasmid (Figure 5(b), lane 1).

### 3.4. Polyclonal Antibodies Anti-NTHiENO Are Specifics

**Figure 6:**
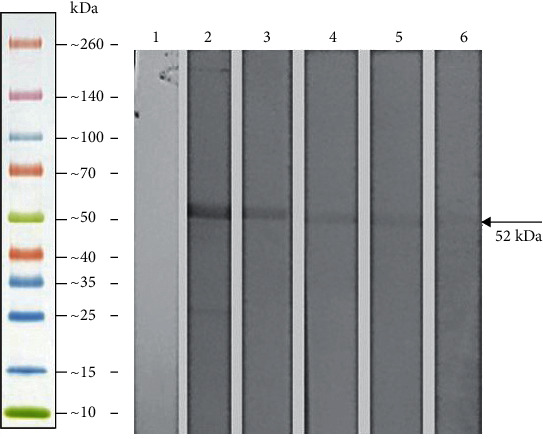
Titer of polyclonal antibodies generated against rNTHiENO. Titration by western blot of the antibodies generated against the recombinant protein. rNTHiENO was migrated on SDS-polyacrylamide gels, and after that, the membranes were electrotransferred to nitrocellulose membranes; as a first antibody, the polyclonal antibody generated at different dilutions was used; as a secondary antibody, a goat anti-rabbit alkaline phosphatase-labeled IgG was used. The image is representative of at least three independent experiments. Lane 1: preimmune serum. Lane 2: dilution 1 : 1000. Lane 3: dilution 1 : 10000. Lane 4: dilution 1 : 20000. Lane 5: dilution 1 : 30000. Lane 6: dilution 1 : 40000. The weight of the recombinant protein is indicated with an arrow.

**Figure 7:**
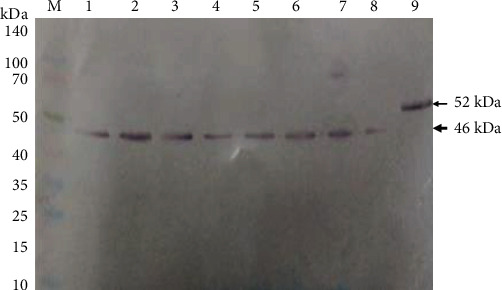
Antibodies anti-rNTHiENO recognize to native enolase of *H. influenzae* typeable and nontypeable. Immunodetection by western blot of the native enolase of *H. influenzae* using anti-rNTHiENO polyclonal antibodies. Lane M: prestained protein marker. Lanes 1-4: total protein extracts of HibBUAPNAN, HibATCC33, HiNTBUAP96, and HiNTBUAPPAU, respectively. Lanes 5-8: enriched membrane proteins, same order. Lane 9: rNTHiENO purified as positive control. The band corresponding to wild enolase (46.0 kDa) in *H. influenzae* strains is indicated with an arrowhead. The band corresponding to rNTHiENO (52 kDa) is indicated with an arrow.

In this study, rabbits were immunized with rNTHiENO to produce polyclonal antibodies. At the end of the immunization scheme, polyclonal anti-rNTHiENO antibodies were quantified and a high titer (1 : 40000) was observed by western blot assay (Figure 6). After that, the serum of these immunized rabbits was used in western blot assays as a first antibody against *H. influenzae* typeable and nontypeable total protein extracts and purified rNTHiENO; these antibodies were capable to detect a band of 52 kDa (Figure 7, lane 9) demonstrating a strong reactivity and antigenicity of rNTHiENO. Moreover, anti-rNTHiENO polyclonal antibodies specifically recognized an approximately 46 kDa band in all total protein extracts of *H. influenzae* (Figure 7, lanes 1-8), which corresponded to the predicted native size of *H. influenzae* enolase and shows a good specificity.

**Figure 8:**
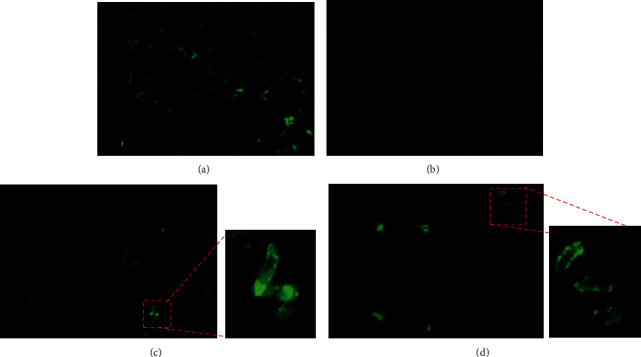
Enolase is exposed in surface in *H. influenzae* typeable and nontypeable. Immunodetection by immunofluorescence indirect (IFI) in nonpermeabilized *H. influenzae* typeable and nontypeable strains was realized. Anti-rNTHiENO antibodies were used as a first antibody; anti-rabbit FITC-labeled IgG was used as a second antibody. Each image is a representative of at least three independent experiments and captured by a fluorescence microscope. (a) *H. influenzae* nontypeable (HiBUAP96) with anti-*H. influenzae* polyclonal antibodies as a positive control. (b) *H. influenzae* nontypeable (HiBUAP96) with preimmune sera as a negative control. (c) *H. influenzae* nontypeable (HiBUAP96) with anti-rNTHiENO polyclonal antibodies; the inset shows a digital amplification (300%) carried out with the ZEISS ZEN lite 2.1 program in order to appreciate the recognition of anti-NTHiENO antibodies in the HiBUAP96 membrane. (d) *H. influenzae* type b (HiBUAPNAN) with anti-rNTHiENO polyclonal antibodies; the inset shows a digital amplification (300%) carried out with the ZEISS ZEN lite 2.1 program in order to appreciate the recognition of anti-NTHiENO antibodies in the HiBUAPNAN membrane.

The antibodies generated against rNTHiENO were also used in immunofluorescence assays against typeable (Hib) and nontypeable *H. influenzae* strains. In nonpermeabilized bacteria, the labeling was found in the membrane of both strains, while intense fluorescence is observed in NTHi and also observed spots of fluorescence on Hib strains. This localization might facilitate its recognition by the host's immune system, suggesting that NTHiENO could have strong antigenic properties (Figure 8).

## 4. Discussion

*H. influenzae* is a bacterium with medical importance because it is the cause of a great variety of local and systemic infections, which mainly affects children under 5 years, adults over 65 years, and immunocompromised individuals [19]. In 1989, the vaccine against *H. influenzae* serotype b was introduced, which has been very successful [20–22]. However, it does not protect against the rest of the serotypes or against nontypeable strains. Recent studies have reported an increase in the incidence of invasive infections caused by non-Hib strains [8]. A study conducted in Bogotá, Colombia, in the period of 2002-2013 was isolated to different strains of *H. influenzae* from invasive diseases. 50.5% were of patients with meningitis, 23.5% of pneumonias, 19.5% of sepsis and bacteremia, 2.0% of others, and 4.5% without data, where the predominant serotype was Hib (40.5%), followed by HiNT (38.0%), Hia (17.5%), Hid (2.0%), Hif (1.5%), and Hie (0.5%) [23]. It is important to mention that continuous surveillance is essential for all *H. influenzae* infections, since non-b serotypes and nontypeable strains can become the main cause of invasive diseases [24].

The lack of a vaccine that generates protection against all serotypes and nontypeable strains promotes the search for new antigens with immunogenic potential against this bacterium.

On the other hand, due to biotechnological approach, the massive sequencing of the genome of multiple microorganisms and the design of bioinformatics tools have given way to the use of reverse vaccinology, a recent approach and first described by Rappuoli in early 2000 [25, 26]. This method is used to predict antigens through the development of genomics and bioinformatics tools [27].

Enolase is an essential enzyme in the metabolism of glucose in different organisms [28]; however, the presence of this protein has been demonstrated on the cell surface of some microorganisms such as bacteria, parasites, and yeasts, where it is found as a plasminogen receptor, and moreover, it has also been shown to be an antigen capable of generating a specific and protective immune response that leads to the neutralization of an infection [13, 15, 29–33].

Due to the above discussion, in this work, we studied the presence of enolase on surface of *H. influenzae* and if NTHiENO could be immunogenic and even more, and if it could generate cross immunogenicity for the typeable and nontypeable *H. influenzae* strains.

Initially, the cloning, expression, and *in silico* characterization of the enolase of NT*H. influenzae* were performed by molecular and immunological techniques. The results demonstrated the rNTHiENO recombinant protein has an approximate weight of 52 kDa fused to His-tag (Figure 5(a), lane 5), and this band was identified by western blot, using anti-His_6X_ monoclonal antibodies (Figure 5(b), lane 2). However, to identify the wild enolase in several *H. influenzae* strains (NTHi and Hib) in total protein extracts, a band with an approximate weight of 46.30 kDa was resolved (Figure 7, lanes 1-8). These results coincide with the enolase weights reported for *V. parahaemolyticus* (48 kDa) [15], *S. iniae* (47 kDa) [13], *C. albicans* (47 kDa) [34], *T. cruzi* (46.01 kDa) [35], and *A. actinomycetemcomitans* (47 kDa) [36].

On the other hand, a multiple alignment of the enolase of different species was performed, in which the expression of enolase in the cell surface has been reported, functioning as a receptor to plasminogen (Figure 2 and Table 1).

It is important to highlight that the enolase of the HiNTBUAP96 and HibBUAPNAN strains has a 99.54% identity; this result indicates that the change between the amino acid sequence of the typable and nontypable strains is not significant. The bacterial enolase with the highest identity with NTHiENO is *V. parahaemolyticus* (85%), and NTHiENO has the lowest identity with human enolases (50%). With the rest of the species, it has a 24-57% identity. Also were identified are the essential residues for its activity, such as the catalytic site and magnesium-binding residues, which determine the necessary conformation for dimerization and enzymatic function as well as the signature of enolase. These motifs coincide with reported by different authors [13, 28, 32, 35].

Plasminogen is a 92 kDa proenzyme; the N-terminal region of plasminogen contains five kringle domains of 80 amino acids, which contain lysine-binding sites [37, 38]; interaction with lysine leads to a conformational change, which makes it more susceptible to cleavage by plasminogen activators (PAs) [39].

An internal motif responsible for plasminogen binding that is exhibiting affinity for kringle domains has been identified in enolase of multiple organisms such as *V. parahaemolyticus* [15], *Streptococcus mutans* [40], *Streptococcus iniae* [13], *Leishmania mexicana* [41], and *Trypanosoma cruzi* [35]. A predicted plasminogen-binding motif (_252_FYNKENGMY_260_) was identified in the enolase sequence of *H. influenzae*; this motif showed a 55.5% similarity with the nine amino acid motifs detected in enolase of *S. pneumoniae* (FY*DKE*R*K*VY; the crucial residues for plasminogen binding are shown in italics). It is reported that point mutations of these amino acids greatly decrease the binding to plasminogen [30, 40]. Of the three important amino acids, Lys255 and Glu256 are conserved in enolase sequence of *H. influenzae*. These data allow us to assume that HiENO could interact with human plasminogen through this putative motif identified in its sequence and that Lys 255 and Glu256 could be important residues to mediate this interaction, which has already been well characterized in *S. pneumoniae*.

On the other hand, the expression of exRNA has been demonstrated in important biological fluids as the blood, semen, saliva, vaginal secretions, and menstrual blood [42]. In *S. pneumoniae*, it has been demonstrated that enolase sequence presents six important binding motifs to exRNA, and moreover, these motifs promote adhesion to endothelial and epithelial cells [18]. In *Helicobacter pylori* enolase, it was also demonstrated the presence of five motifs conserved that might interact with host-derived exRNA [43]. In this study, the NTHi enolase presents five of these motifs; this could indicate that *H. influenzae* use these motifs to have adhesion to exRNA, thus contributing to the colonization and dissemination of bacteria; however, more assays must be done to verify this assumption.

One of the aims of this work was to demonstrate if enolase is expressed on the cell surface of *H. influenzae*. Since enolase is described as a nonclassical export protein due to the lack of signal peptide [44], possible transmembranal helices were located in the enolase sequence by *in silico* analysis. In these results, a transmembranal (TM) domain is identified in the region comprised by amino acids Asn_105_-Ala_124_ (Supplementary Figure 1). These results allow inferring that the enolase of *H. influenzae* could be anchored in the bacterial membrane through this region. A group of researchers in 2011 identified an alpha helix with hydrophobic characteristics in the enolase of *Bacillus subtilis* in the position Ala_108_-Leu_126_ and within this region a domain TM (Ala_110_ to Cys_118_) and may be immersed in the membrane of *B. subtilis* [45]. In *T. cruzi*, a transmembrane domain was also identified in the N-terminal region between the amino acids Gly_105_-Ala_122_ [35]; these latter data agree with those obtained for enolase from *H. influenza*e.

Although the precise mechanism by which enolase is exported to the surface is unknown, one hypothesis is that it was carried out by micromaterials; however, it was observed that transport through vesicles is not significant, so there must be alternative mechanisms to release this protein lacking signal peptide to the medium. On the other hand, it has been observed that this alpha helix is essential for their exporting, since when that region is mutated (Ala_108_-Leu_126_) and replaced by another alpha helix (so as not to alter the three-dimensional structure); note that the enolase of *B. subtilis* is no longer secreted into the environment [45].

On the other hand, the immunogenic properties of NTHiENO were analyzed using a bioinformatics approach. When the protein was analyzed with the VaxiJen program, it showed that enolase could function as a protective antigen. In turn, along the enolase sequence, 10 epitopes were predicted for B cells and 13 for CTL cells, and four of them showed overlap. In this regard, previous reports confirm that stimulation with recombinant enolase from *Clonorchis sinensis* (rCs enolase) leads to the production of IgG1 and IgG2a and that rCs enolase fused with a *Bacillus subtilis* spore induces Th1/Th2 immune responses [46]. On the other hand, *C. albicans* enolase can stimulate the secretion of IL-12 and IL-10, in addition to protective antibodies IgG1 and IgG2a isotype [47]. Another study showed that recombinant enolase from *T. cruzi* was recognized by sera from infected patients; this indicates that enolase is recognized by the immune system of the patients and therefore is immunogenic [35]. The use of enolase to trigger a protective immune response was reported in a study carried out by Wang et al. in 2015, where they observed the participation of enolase in adhesion and invasion processes by *Streptococcus iniae* in BHK-21 cells (hamster kidney cancer cells); furthermore, immunization with the recombinant protein can confer effective protection against *S. iniae* infection in mice [13]. Another study also reports that *S. sobrinus* rEnolase acts as an immunogenic antigen, observing a correlation between previous immunization of recombinant enolase and decreased oral colonization by *S. sobrinus*, in the rat model [48].

Further, in *Vibrio parahaemolyticus*, it was shown that vaccination with recombinant enolase confers effective immunity against a lethal dose of *V. parahaemolyticus* in a mouse model [15]. The protective effect of enolase has also been observed in parasites. For example, a study conducted in India in 2007 showed that *Plasmodium falciparum* enolase is present on the surface of merozoites, and the use of anti-*P. falciparum* enolase antibodies inhibits its growth *in vitro* culture; in addition, it was also observed that mice immunized with r-Pfen demonstrated protection against the lethal strain *Plasmodium* yoelii 17XL [49]. Similar results were reported for *Ascaris suum* enolase, where a decrease of 61.13 and 88.62% is observed in the recovery of larvae and eggs, from mice previously immunized with recombinant enolase and later experimentally infected with 2500 larvae of *A. suum*; suggesting once again, enolase is a vaccine candidate against *A. suum* [50]. On the other hand, it has been reported that enolase is involved in cancer, systemic fungal disease, and dental diseases [11], and it has even been reported that the expression of enolase on the surface of lung cancer cells promotes its migration or metastasis [51, 52]. Given its importance in different pathological events, enolase has been described as an immunodominant antigen, considered as a potential vaccine candidate [34, 50] in fungi, bacteria, and parasites, and as therapeutic targets against cancer.

The above studies allow us to propose *H. influenzae* enolase as a possible immunogen to be studied in the development of a vaccine, also taking into account the *in silico* analysis which evidences the immunogenic characteristics of this protein.

Finally, immunofluorescence assays showed that enolase is present on the cell surface in both nontypeable (noncapsulated) and typeable (capsulated) *H. influenzae* strains, although the recognition pattern was different, homogeneous in the nontypeable and in the form of spots in the typeable, probably due to the presence of the capsule that interferes with an adequate anchorage by the enolase. However, these results demonstrate in an important way the presence of the enolase on the bacterial surface, and in addition, the antibodies generated specifically recognize this protein.

These results are in agreement with those described in other bacterial models, where the presence of enolase on the bacterial surface has been described such as *Streptococcus pneumoniae*, *Streptococcus pyogenes*, *Staphylococcus aureus*, *Mycoplasma fermentans*, *Pseudomonas aeruginosa*, and *Mycobacterium tuberculosis* [29, 31, 53–55]. Our results indicate that the surface enolase of a bacterium could be a key molecule in pathogenesis by facilitating bacterial interaction with host cells, causing invasion of host tissue.

## 5. Conclusion

The present study demonstrates that specific polyclonal anti-rNTHiENO antibodies can recognize both typeable and nontypeable strains of *H. influenzae*. Furthermore, the results obtained in this work suggest that immunization with rNTHiENO will be able to generate a good immunogenic response and could provide protection against experimental infection caused by capsulated and noncapsulated strains of *H. influenzae*. In addition, we have demonstrated for the first time, to our knowledge, that the *H. influenzae* enolase protein is located on the cell surface. The immunogenic properties evidenced by *in silico* analysis and the surface localization of enolase are important features to consider *H. influenzae* enolase as a potential candidate for vaccine development; however, further studies are needed to confirm that enolase of *H. influenzae* could be a good vaccine candidate.

## Figures and Tables

**Table 1:** Biochemical characteristics of NT*H. influenzae* enolase.

Feature	*H. influenzae*	*S. pneumoniae*	*H. sapiens*
Mg^2+^	D_244_	D_242_	S_40_
	E_291_	E_291_	D_245_
	D_318_	D_318_	E_293_
			D_318_
Catalytic sites	H_159_	H_155_	H_158_
	E_168_	E_164_	E_167_
	E_209_	E_205_	E_210_
	D_318_	E_291_	E_293_
	K_343_	D_318_	D_318_
	R_372_	K_343_	K_343_
	S_373_	R_372_	R_372_
	K_394_	S_373_	S_373_
		K_394_	K_394_
RNA binding (%)^a^	_58_RFLGKGVLK_66_ (44%)	_58_RYGGLGTQK_66_	_56_RYMGKGVSK_64_
	_103_KSNFGA_108_ (50%)	_103_KGKLGA_108_	_103_KSKFGA_108_
	_191_HNLAKVLKSKGMSTA_205_ (60%)	_187_HALKKILKSRGLETA_201_	_190_HNLKNVIKEKYGKDATN_206_
	_310_GDRVQL_315_ (66.67%)	_310_GKKVQL_315_	_312_GIQV_315_
	_400_RSDRIAK_406_ (85.71%)	^400^RTDRIAK^406^	^400^RSERLAK^406^
		_432_LKK_434_	_432_LAK_434_
Enolase signature	_340_ILIKFNQIGSLTET _353_	_340_ILIKVNQIGTLTET_353_	_340_LLLKVNQIGSVTES_353_
Plasminogen binding (%)^b^	_252_FYNKENGMY_260_ (55.5%)	_248_FYDKERKVY_256_	_251_FFRSGKY_257_

^a^Homology percent with respect to exRNA-binding sites in *S. pneumoniae* [17]. ^b^Homology percent with respect to plasminogen-binding site in *S. pneumoniae* [29].

**Table 2:** Cytotoxic T lymphocyte epitopes for different HLA supertypes predicted in NT*H. influenzae* enolase.

Peptide no.	Peptide sequence	HLA super type	Overlapping with B epitope	Proteasomal cleavage sites
1	HLEGGFVGL	A26, B39, B44	No	Yes (2 sites)
2	ASTGSREAL	B7, B39	Yes	Yes (4 sites)
3	RFLGKGVLK	A3, B27, B29	Yes	Yes (4 sites)
4	SNFGANAIL	A2, B39, B58	No	Yes (2 sites)
5	AASKGLPLY	A1, A3, A26, B7, B58, B62	No	Yes (3 sites)
6	ELNGTAGVY	A1, A26, B44, B62	No	Yes (3 sites)
7	GAKTLREAL	B7, B8, B58	No	Yes (3 sites)
8	EVFHNLAKV	A26, B8, B44	No	Yes (4 sites)
9	EAVEKAGYV	A26, B7, B39	No	Yes (3 sites)
10	YVLGKDUTL	A2, B8, B39, B62	No	Yes (5 sites)
11	FTSQEFTHY	A1, A26, B39, B44, B58, B62	Yes	Yes (4 sites)
12	ESDWEGFAY	A1, A26, B44, B58	Yes	Yes (2 sites)
13	LTETLAAIK	A1, A2, A3, B44	No	Yes (4 sites)

## Data Availability

All data are available from the corresponding author on reasonable request.

## Supplementary Materials

Supplementary Materials Supplementary 1. Figure S1: NTHiENO transmembrane helix prediction. In order to predict the presence of a transmembrane helix in the enolase sequence, three bioinformatics programs were used: PredictProtein, TMpred, and HMMTOP; these showed very similar results, the presence of a transmembrane helix in the HiENO sequence, and the results obtained in the HMMTOP program are presented. This program predicts the presence of the transmembrane helix from position Asn_105_ to Ala_124_ (highlighted in red); it also shows an intracellular or cytoplasmic region Met_1_-Ser_104_ and the extracellular region Ser_125_-Tyr_437_.
