# Supplementary material for: Cloning and Characterization of Immunological Properties of Haemophilus influenzae Enolase
Source: J Immunol Res. 2021 Jun 16;2021:6629824. doi: 10.1155/2021/6629824 (PMC8225457; doi:10.1155/2021/6629824)

**Supplementary figure 1. NT HiENO Transmembrane Helix Prediction**

In order to predict the presence of a transmembrane helix in the enolase sequence, three bioinformatics programs were used PredictProtein, TMpred and HMMTOP, these showed very similar results, the presence of a transmembrane helix in the HiENO sequence, the results obtained in the HMMTOP program are presented. This program predicts the presence of the transmembrane helix from position Asn_105_ to Ala_124_ (highlighted in red), it also shows an intracellular or cytoplasmic region Met_1_-Ser_104_ and the extracellular region Ser_125_-Tyr_437_.


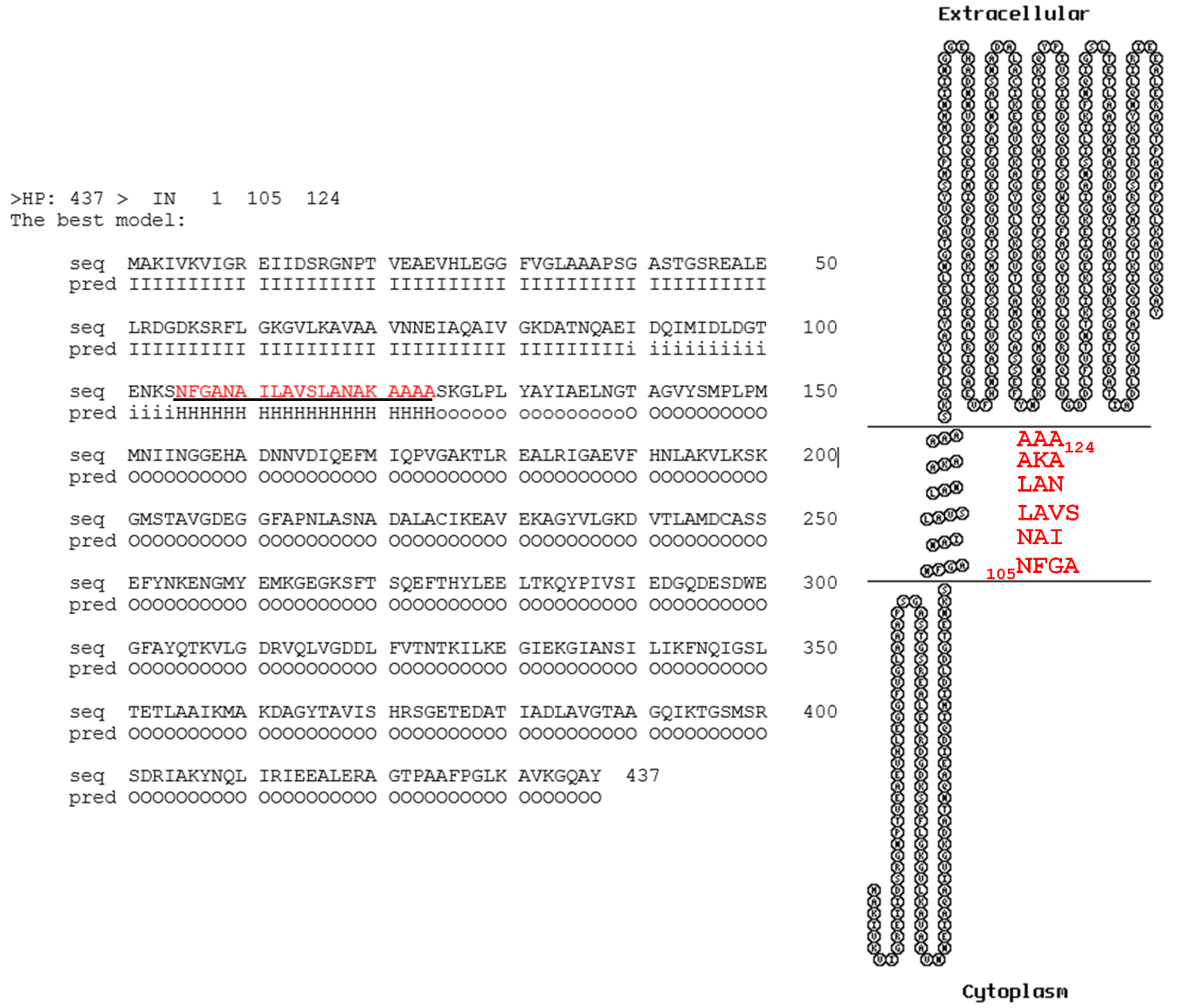

Supplement: Supplementary Materials — Supplementary 1. Figure S1: NTHiENO transmembrane helix prediction. In order to predict the presence of a transmembrane helix in the enolase sequence, three bioinformatics programs were used: PredictProtein, TMpred, and HMMTOP; these showed very similar results, the presence of a transmembrane helix in the HiENO sequence, and the results obtained in the HMMTOP program are presented. This program predicts the presence of the transmembrane helix from position Asn105 to Ala124 (highlighted in red); it also shows an intracellular or cytoplasmic region Met1-Ser104 and the extracellular region Ser125-Tyr437. [file 6629824.f1.docx]
